# Impact of Delayed Adjuvant Radiotherapy in the Survival of Women with Breast Cancer

**DOI:** 10.7759/cureus.3071

**Published:** 2018-07-30

**Authors:** Christian H Flores-Balcázar, Lourdes Flores-Luna, Cynthia Villarreal-Garza, Aida Mota-García, Enrique Bargalló-Rocha

**Affiliations:** 1 Radiation Oncology, National Institute of Nutrition and Medical Sciences, Mexico City, MEX; 2 Epidemiology, National Institute of Public Health, Cuernavaca, Morelos, MEX; 3 Oncology, National Cancer Institute, Mexico City, MEX; 4 Radiotherapy, National Cancer Institute, Mexico City, MEX; 5 Surgical Oncology, National Cance, Mexico City, MEX

**Keywords:** breast cancer, delay, adjuvant radiotherapy, survival

## Abstract

Objective

Our objective was to determine whether a delay in adjuvant radiotherapy is related to a decrease in relapse-free survival and disease-specific survival of women with operable breast cancer.

Methods

Data on 1000 patients diagnosed with breast cancer were recorded. The cohort was divided into five groups according to the timing of radiotherapy: ≤30 days, 31 to 60 days, 61 to 90 days, 91 to 120 days, and >120 days. The relapse-free survival and disease-specific survival were also calculated in relation to the number of patients.

Results

This study found no statistical difference for delays in adjuvant radiotherapy in patients with early breast cancer, but we noted a statistical decrease in disease-specific survival in patients with locally advanced breast cancer receiving radiotherapy after a delay of at least 60 days.

Conclusion

Waiting times for radiotherapy should be as short as reasonably achievable, given the specific risk factors in the individual patient.

## Introduction

In recent decades, breast cancer has become one of the leading causes of cancer-related death in women worldwide. Up to 50% of breast cancer diagnoses and 60% of deaths due to this cancer occur in women living in middle-income countries [1]. The incidence and mortality rates in these countries are higher due to limited medical infrastructure and a lack of promotion of self-examination and cancer prevention information. This situation is of special concern for hospitals in developing countries due to increased waiting lists for access to treatment [2-3]. Because radiation therapy has a high impact on the treatment of breast cancer by avoiding mastectomy in patients with early tumors and diminishing local relapse in patients with breast-conserving surgery and mastectomy, waitlists for treatment initiation have grown dramatically worldwide [4-6]. Unfortunately, access to radiation therapy is still difficult in most of Latin America and Caribbean countries. The Lancet Radiotherapy Commission report published in 2015 estimated that premature mortality due to a lack of optimal radiation therapy over the next 20 years in low and middle-income countries would result in a billion lives lost [7].

Delays between surgery and adjuvant radiotherapy may be due to a lack of linear accelerators and trained personnel. However, existing knowledge regarding adjuvant radiotherapy delay after surgery is poor. An overview of studies from the National Surgical Adjuvant Breast and Bowel Project group [8] stated that local recurrence is an important prognosticator of survival. Therefore, the impact of delayed radiotherapy on local recurrence could theoretically also affect survival. In a study from the Surveillance, Epidemiology, and End Results-Medicare group, 13,907 women aged over 65 years were assessed. Ninety-seven percent of patients initiated adjuvant irradiation in three months. For these patients, a delay in radiotherapy delivery of up to three months was not associated with a decreased survival. However, patients who received radiotherapy after three months had higher overall mortality (hazard ratio (HR), 1.92) and cancer-specific mortality (HR, 3.84), and a 90% increase in overall mortality rate was observed, which corresponds to a four-fold increased likelihood of dying from breast cancer [9]. A delay in delivering efficient radiotherapy seems to be related to an increased risk of local recurrence [10].

The effect of treatment delay on outcomes cannot easily be investigated in randomized trials. Therefore, observational studies based on high-quality routinely recorded data are important. This study aimed to determine whether a delay in adjuvant radiotherapy is related to a decrease in relapse-free survival (RFS) and disease-specific survival (DSS) of women with operable breast cancer comprising a retrospective cohort at the National Cancer Institute in Mexico City.

## Materials and methods

This retrospective study consisted of a random selection of 1000 of 1610 women who met the inclusion criteria and were treated at the National Cancer Institute of Mexico City from 2005 to 2012.

Data collection and variable definition

For each patient, clinical data were obtained retrospectively from the patient’s medical records. Data on 1000 patients diagnosed with breast cancer and treated at the Department of Radiotherapy of the National Cancer Institute, Mexico City, were recorded. The cohort was divided into five groups according to the timing of radiotherapy following surgery: ≤30 days, 31 to 60 days, 61 to 90 days, 91 to 120 days, and >120 days. The cohort included women over 18 years of age with a histopathological diagnosis of ductal and lobular infiltrating cancer with accurate dates of diagnostic and initial treatments, who had an initial grading of clinical stage I-IIIB according to the Tumor, Node, Metastasis (TNM) classification system. The following patients were excluded: men with breast cancer, women with in situ breast carcinoma, women whose surgery was performed outside our institution, patients with tumors with features of inoperability by the Haagensen criteria (i.e., extensive breast edema, satellite nodules in the skin, inflammatory cancer, parasternal tumor nodules, confirmed supraclavicular metastases, arm edema, and distant metastases), women with synchronous or metachronous breast cancer, women with a history of any other type of cancer except non-melanoma skin cancer, women in whom the standard treatment has not been granted due to comorbidities that put lives at risk (i.e., morbid obesity, heart disease, and renal failure) and women with metastatic disease at diagnosis. Patients with institutional records who had not completed treatment at our hospital or whose initial treatment took more than six months for any reason were also excluded. The study was approved by the ethics committee as required by both Health Institutes, the National Institute of Public Health, and the National Cancer Institute.

Systemic treatment

During the study, trends for administering neoadjuvant and adjuvant systemic therapy in breast cancer varied. Twelve years ago, the number of tumor-positive axillary lymph nodes, large tumor size, high malignancy grade, and young age were indications for adjuvant chemotherapy. Over time, the use of neoadjuvant chemotherapy has increased in an attempt to diminish the burden of disease and achieve breast-conserving surgery. Also, the types of chemotherapy and hormone therapy alone or in combination have evolved.

Radiotherapy scheme

Almost all patients received 50 Gy to the whole breast, delivered in 2-Gy fractions five times per week by a tangential field technique in women with early breast cancer. This was followed by a boost of 10 Gy to 16 Gy to the primary tumor bed in 2-Gy fractions five times per week using electron beam therapy. Women with locally advanced cancer were irradiated with a supraclavicular field at the same dose as the tangential fields when indicated.

Definition of timing

Patients referred to our department by the surgeon or the oncologist were scheduled for treatment. The timing of radiotherapy was defined as the number of days from surgery to the start of irradiation. The timespan ranged from 11 days to 496 days following surgery. No selection in starting radiotherapy was made on any known prognostic factor.

Statistical analysis

Measures of dispersion and central tendency were used for analysis. Chi-squared tests and Fisher’s exact tests were used to assess inter-group differences for categorical data. Determination of survival at five years for demographic and clinical characteristics was performed. Survival statistics were acquired based on the number of patients and calculated Kaplan-Meier estimates. The surgery date, defined as the date of the last surgery on the breast, was used as the start of observation, and the date of the last medical follow-up visit was used as the end of the follow-up period. The timing of radiotherapy was calculated as the interval between surgery and the radiotherapy start date and defined as the date in which the first fraction of radiotherapy was administered.

The RFS and DSS, corrected for intercurrent death, were also calculated in relation to the number of patients. This means that data on patients who died of other causes were regarded as censored data. Censoring variables were defined by women who did not report events of interest within the study period, and as censorship criteria included: a) death due to a different cause than breast cancer; b) loss during the follow-up (e.g., address not notified, refusal to continue in the study, lack of follow-up treatment), and c) all those women who remained alive until the end of the study period. For comparison of survival distributions, the log-rank test was used. Variables that were univariately related to the outcomes of interest (P <0.05) were entered in the multivariate analyses. We used Cox proportional hazards modeling to test for the independent effect of radiotherapy timing after adjusting for known prognostic factors, and HR estimated with 95% confidence limits are presented. A test for trend across the five ordered quintiles was performed. The quintile with the smallest timing, <30 days, was the referent group. All analyses were performed using Stata Statistical Software, Release 14 (StataCorp LP, College Station, TX).

## Results

The median time of follow-up was 5.74 years (range, six months to 11.5 years). The median waiting time from surgery to the start of adjuvant radiotherapy was 152 days (range, 11 to 496 days). Reasons for the delay in starting adjuvant radiotherapy were related to arm limitation in patients with radical mastectomy, postoperative wound-healing complications, delay in referral to our department, and waitlist size for starting the irradiation. The mean patient age was 49 years (range, 24 to 86 years). The tumor and patients characteristics according to the quintiles are shown in Table 1. The quintiles showed a significant difference for hormone status, her2Neu expression, stage, type of surgery, administration, and timing of chemotherapy were statistically significant (p <0.001, p <0.001, p <0.038, p <0.001, and p <0.001, respectively).

**Table 1:** Distribution of Patients on the Basis of Clinicopathological Features and Time Delay *Chi-squared test/Fisher’s test. **Status of the patient by the time the statistical analysis was done. LVP: lymphovascular permeation.

Variable	<30d	31-60d	61-90d	91-120d	>121d	Total (%)	p Value*
	14	97	255	159	475	1000	
Age (years)							
<50	5 (0.83)	59 (9.83)	157 (26.17)	96 (16.00)	283 (47.17)	600 (100)	0.437
>50	9 (2.25)	38 (9.50)	98 (24.50)	63 (15.75)	192 (48.00)	400 (100)	
Hormonal Status							
Premenopausal	5 (1.20)	42 (10.07)	106 (25.42)	67 (16.07)	197 (47.24)	417 (100)	0.988
Postmenopausal	9 (1.54)	55 (9.43)	149 (25.56)	92 (15.78)	278 (47.68)	583 (100)	
Grade							
Low	1 (0.55)	20 (11.05)	43 (23.76)	20 (11.05)	97 (53.59)	181 (100)	0.348
Moderate	7 (1.98)	27 (7.63)	90 (25.42)	60 (16.95)	170 (48.02)	354 (100)	
High	6 (1.45)	42 (10.12)	112 (26.99)	71 (17.11)	184 (44.34)	415 (100)	
Not Available	0 (0)	8 (16.0)	10 (20.0)	8 (16.0)	24 (48.00)	50 (100)	
Histology							
Ductal	13 (1.43)	81 (8.89)	235 (25.80)	147 (16.14)	435 (47.75)	911 (100)	0.099
Lobular	1 (1.12)	16 (17.98)	20 (22.47)	12 (13.48)	40 (44.90)	89 (100)	
Estrogen Receptor							
Positive	8 (1.21)	64 (9.67)	158 (23.87)	108 (16.31)	324 (48.94)	662 (100)	<0.001
Negative	6 (1.78)	33 (9.76)	97 (28.70)	51 (15.09)	151 (44.67)	338 (100)	
Progesterone Receptor							
Positive	5 (0.85)	54 (9.17)	133 (22.58)	86 (14.60)	311 (52.80)	589 (100)	<0.001
Negative	9 (2.19)	43 (10.46)	122 (29.68)	73 (17.76)	164 (39.90)	411 (100)	
Her2Neu Expression							
Positive	5 (2.54)	25 (12.69)	60 (30.46)	28 (14.21)	79 (40.10)	197 (100)	0.038
Negative	9 (1.12)	72 (8.97)	195 (24.28)	131 (16.31)	396 (49.32)	803 (100)	
PVL							
Yes	7 (0.85)	15 (8.72)	37 (21.51)	27 (15.70)	86 (50.00)	172 (100)	0.015
No	7 (4.07)	82 (9.90)	218 (26.33)	132 (15.94)	389 (46.98)	828 (100)	
Molecular Profile							
Luminal A	7 (1.11)	58 (9.22)	149 (23.69)	99 (15.74)	316 (50.24)	629 (100)	0.326
Luminal B	1 (1.14)	9 (10.23)	21 (23.86)	14 (15.91)	43 (48.86)	88 (100)	
Her2Neu	3 (3.16)	13 (13.68)	31 (32.63)	11 (11.58)	37 (38.95)	95 (100)	
Triple Negative	3 (1.60)	17 (9.04)	54 (28.72)	35 (18.62)	79 (42.02)	188 (100)	
TNM Stage							
I	0 (0)	10 (9.17)	27 (24.77)	11 (10.09)	61 (55.96)	109 (100)	<0.001
IIA	3 (1.92)	8 (5.13)	22 (14.10)	20 (12.82)	103 (66.03)	156 (100)	
IIB	3 (1.29)	27 (11.59)	59 (25.32)	35 (15.02)	109 (46.78)	233 (100)	
IIIA	4 (1.18)	40 (11.83)	108 (31.95)	74 (21.89)	112 (33.14)	338 (100)	
IIIB	4 (2.44)	12 (7.32)	39 (23.78)	19 (11.59)	90 (54.88)	164 (100)	
Surgery Type							
Breast Conserving	2 (0.84)	23 (9.70)	49 (20.68)	29 (12.24)	134 (56.54)	237 (100)	0.023
Mastectomy	12 (1.57)	74 (9.70)	206 (27.00)	130 (17.04)	341 (44.69)	763 (100)	
Chemotherapy							
Yes	13 (1.43)	83 (9.15)	220 (24.26)	145 (15.99)	446 (49.17)	907 (100)	0.009
No	0 (0)	12 (13.95)	34 (39.53)	11 (12.79)	29 (33.72)	93 (100)	
Timing of Chemotherapy							
Neoadjuvant	11 (1.99)	67 (12.09)	174 (31.41)	102 (18.41)	200 (36.10)	554 (100)	<0.001
Adjuvant	2 (0.56)	15 (4.24)	48 (13.56)	43 (12.15)	246 (69.49)	354 (100)	
None	1 (1.09)	15 (16.30)	33 (35.87)	14 (35.87)	29 (31.52)	92 (100)	
Hormone Therapy							
Yes	12 (1.60)	69 (9.19)	188 (25.03)	117 (15.58)	365 (48.60)	751 (100)	0.578
No	2 (0.80)	28 (11.24)	67 (26.91)	42 (16.87)	110 (44.10)	249 (100)	
Recurrence							
Yes	5 (2.09)	29 (12.13)	58 (24.27)	37 (15.48)	110 (46.03)	239 (100)	0.0500
No	9 (1.18)	68 (8.94)	197 (25.89)	122 (16.03)	365 (47.96)	761 (100)	
Status**							
Alive	9 (1.18)	68 (8.54)	197 (25.89)	122 (16.03)	365 (47.96)	761 (100)	0.549
Dead	5 (2.09)	29 (12.13)	58 (24.27)	37 (15.48)	110 (46.03)	239 (100)	

Relapse-free survival

Overall, we found 239 relapses (23.9%), five in the first group (<30 days), 29 in the second group (31 to 60 days), 58 in the third group (>61 days), 37 in the fourth group (>91 days), and 110 in the fifth group (>121 days). The median time between surgery and the occurrence of relapse was 2.8 years (interquartile range, 0.1 to 9.97).

**Figure 1:**
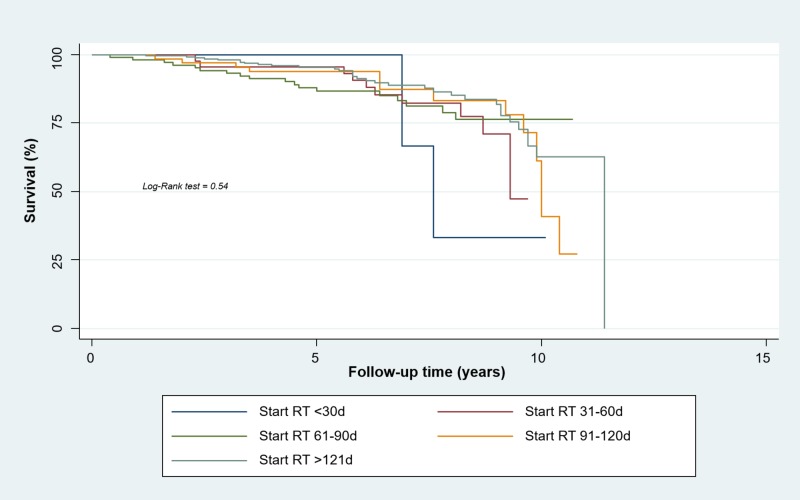
Relapse-free Survival of Women with Early Breast Cancer

For early-stage cancers (TNM Stages I-IIB), the Kaplan-Meier method found a five-year-RFS rate of 100% for the first group, 95.4% for the second group (95% confidence interval (CI), 0.83 to 0.98), 86.7% for the third group (95% CI, 0.78 to 0.92), 93.8% for the fourth group (95% CI, 0.84 to 0.97), and 95.4% for the fifth group (95% CI, 0.92 to 0.97). Even when a trend to relapse was seen in patients irradiated longer than 60 days, no statistically significant relationship between relapse and timing of radiotherapy was found (p = 0.54) (Figure 1).

**Figure 2:**
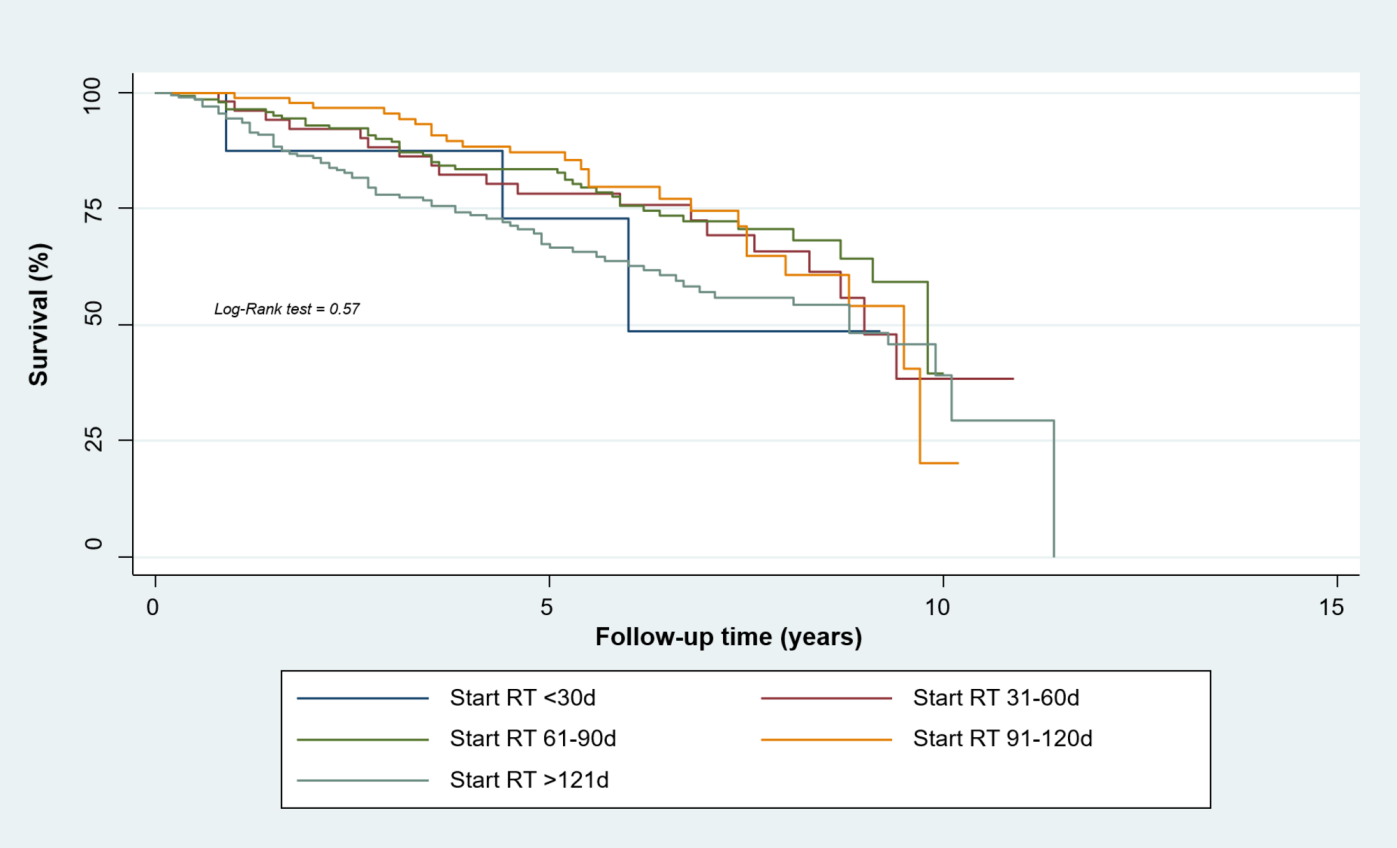
Relapse-free Survival of Women with Locally Advanced Breast Cancer

For locally advanced stages (TNM Stages IIIA-IIIB), the Kaplan-Meier method found a five-year-RFS rate of 73% for the first group (95% CI, 0.28 to 0.93), 78% for the second group (95% CI, 0.64 to 0.87), 82.7% for the third group (95% CI, 0.75 to 0.88), 88.1% for the fourth group (95% CI, 0.79 to 0.93), and 66.9% for the fifth group (95% CI, 0.59 to 0.74). Also, no statistically significant relationship between relapse and timing of radiotherapy was found (p = 0.57) (Figure 2).

When the significant patent variables (e.g., estrogen receptor, progesterone receptor, Her2Neu status, type of surgery, timing of chemotherapy, stage, and lymphovascular permeation) shown in Table 1 were considered in the analysis, the HR for the second group was 0.83 (95% CI, 0.31 to 2.18), 0.67 (95% CI, 0.26 to 1.69) for the third group, 0.69 (95% CI, 0.26 to 1.79) for the fourth group, and 0.91 (95% CI, 0.36 to 2.29) for the fifth group, compared with the first group as shown in Table 2.

**Table 2:** Adjusted Proportional Hazard Regression Results for Relapse-free Survival *Proportional hazards ratio, adjusted by the variables included in the model. HR: hazard ratio; RFS: relapse-free survival.

RFS Variable	HR*
Timing of Radiotherapy (days)	
<30	1.0
31-60	0.83 (0.31-2.18)
61-90	0.67 (0.26-1.69)
91-120	0.69 (0.26-1.69)
>121	0.91 (0.36-2.29)
Stage	
I	1.0
IIA	3.55 (1.22-10.3)
IIB	3.54 (1.22-10.22)
IIIA	6.84 (2.41-19.45)
IIIB	8.10 (2.81-23.39)
Estrogen Receptor Status	
Positive	1.0
Negative	0.89 (0.63-1.26)
Progesterone Receptor Status	
Positive	1.0
Negative	0.89 (0.63-1.25)
Her2Neu Status	
Negative	1.0
Positive	0.87 (0.63-1.21)
Lymphovascular Permeation	
Negative	1.0
Positive	0.83 (0.59-1.17)
Timing of Chemotherapy	
No Chemotherapy	1.0
Neoadjuvant	1.66 (0.74-3.74)
Adjuvant	1.19 (0.53-2.70)

Disease-specific survival

**Figure 3:**
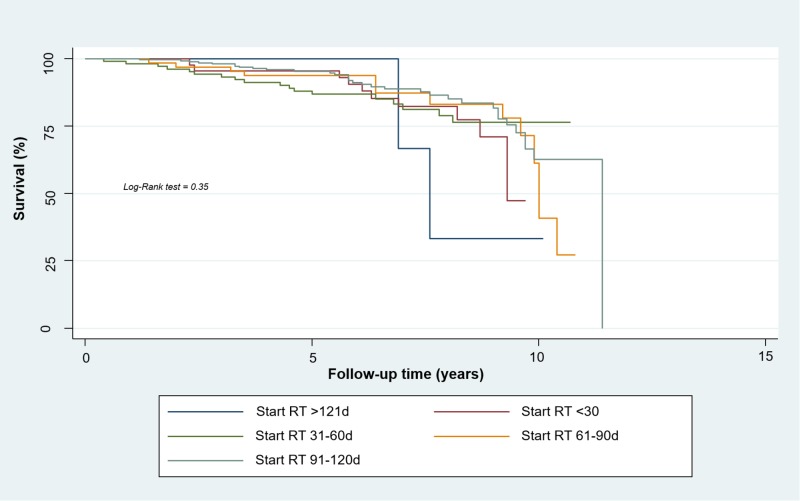
Disease-specific Survival of Women with Early Breast Cancer

Five-year survival for TMN Stage I patients was 91.9% (95% CI, 0.84 to 0.96), 91.1% (95% CI, 0.85 to 0.95) for Stage IIA patients. Five year survival for patients with Stage IIB was 87.9% (95% CI, 0.83 to 0.92), 78.04% (95% CI, 0.73 to 0.82) for Stage IIIA patients, and 69.4% for Stage IIIB patients (95% CI, 0.61 to 0.76). For early stages (TMN Stages I-IIB) the Kaplan-Meier method found a five-year-DSS rate of 100% for the first group, 93.3% for the second group (95% CI, 0.81 to 0.98), 81.8% for the third group (95% CI, 0.73 to 0.88), 92.2% for the fourth group (95% CI, 0.82 to 0.97), and 91.2% for the fifth group (95% CI, 0.87 to 0.94). We found no statistically significant relationship between disease-specific survival and timing of radiotherapy (p = 0.35) (Figure 3).

**Figure 4:**
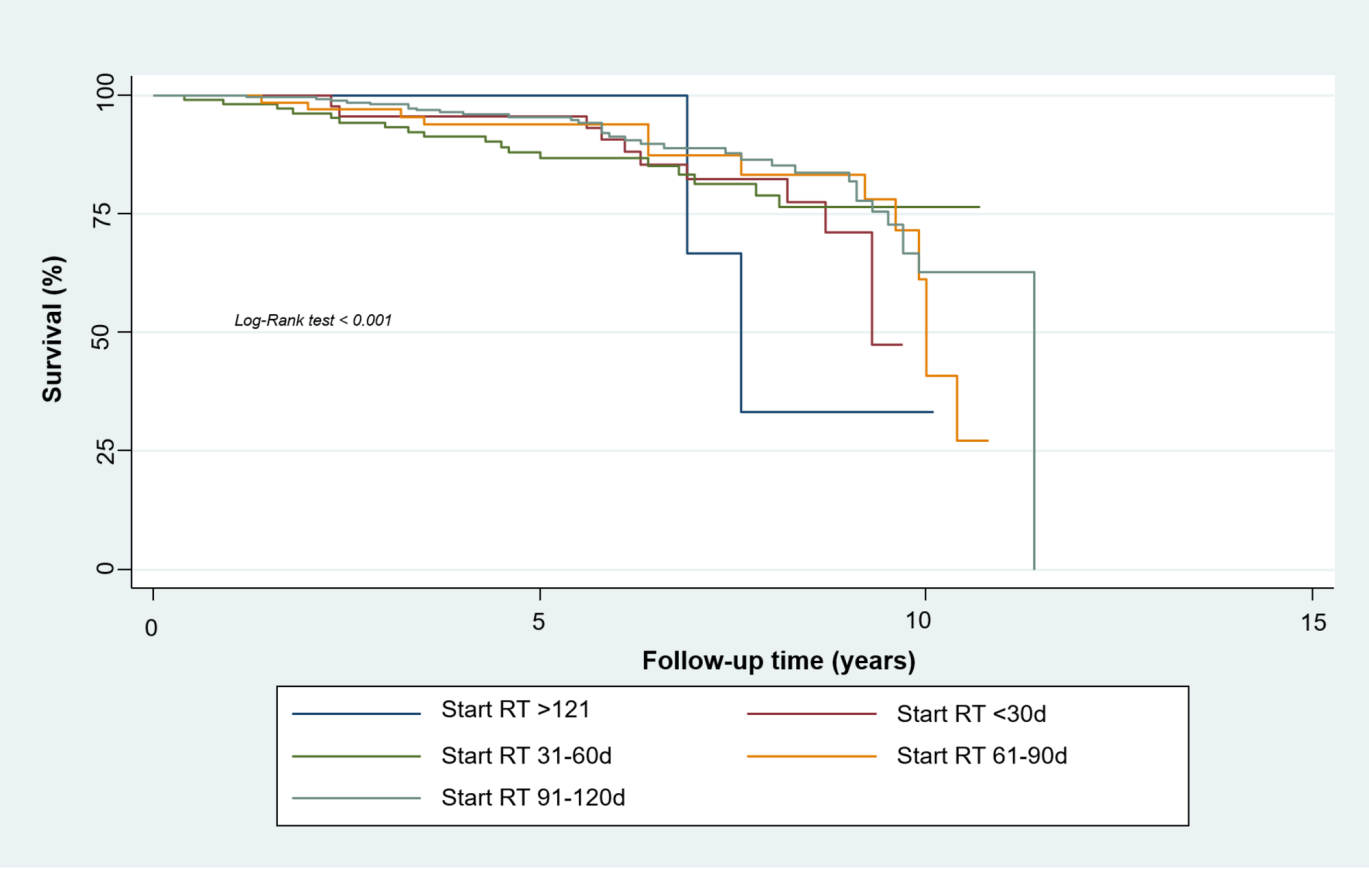
Disease-specific Survival of Women with Locally Advanced Breast Cancer

For locally advanced stages (TMN Stages IIIA-IIIB) the Kaplan-Meier method found a five-year-DSS rate of 62.5% for the first group (95% CI, 0.23 to 0.86), 74.5% for the second group (95% CI, 0.60 to 0.84), 81.1% for the third group (95% CI, 0.74 to 0.87), 88.2% for the fourth group (95% CI, 0.79 to 0.93), and 65.1% for the fifth group (95% CI, 0.57 to 0.72). We found a statistically significant relationship between disease-specific survival and the timing of radiotherapy (p<0.001) (Figure 4).

When the significant patent variables (e.g., estrogen receptor, progesterone receptor, Her2Neu status, type of surgery, timing of chemotherapy, stage, and lymphovascular permeation) shown in Table 1 were considered in the analysis, the HR for the second group was 1.07 (95% CI, 0.37 to 3.14), 1.04 (95% CI, 0.38 to 2.93) for the third group, 0.83 (95% CI, 0.29 to 2.41) for the fourth group, and 1.54 (95% CI, 0.56 to 4.27) for the fifth group, compared with the first group as shown in Table 3.

**Table 3:** Adjusted Proportional Hazard Regression Results for Disease-specific Survival *Proportional hazards ratio, adjusted by the variables included in the model. DSS: disease-specific survival; HR: hazard ratio.

DSS Variable	HR*
Timing of Radiotherapy (days)	
<30	1.0
31-60	1.07 (0.37-3.14)
61-90	1.04 (0.38-2.93)
91-120	0.83 (0.29-2.41)
>121	1.54 (0.56-4.27)
Stage	
I	1.0
IIA	1.18 (0.59-2.38)
IIB	1.29 (0.65-2.56)
IIIA	2.11 (1.09-4.09)
IIIB	2.83 (1.45-5.57)
Estrogen Receptor Status	
Positive	1.0
Negative	0.80 (0.57-1.14)
Progesterone Receptor Status	
Positive	1.0
Negative	0.61 (0.43-0.86)
Her2Neu Status	
Negative	1.0
Positive	1.23 (0.87-1.75)
Lymphovascular Permeation	
Negative	1.0
Positive	0.73 (0.52-1.03)
Timing of Chemotherapy	
No Chemotherapy	1.0
Neoadjuvant	0.69 (0.39-1.22)
Adjuvant	0.37 (0.21-0.67)

## Discussion

The results of this cohort study refine the hypothesis that the timing of adjuvant radiotherapy might have an impact on outcomes. Few data exist evaluating the implications of delayed initiation of postoperative radiotherapy, and, as a result, the optimal timing for starting postoperative irradiation is not yet well defined in early or locally advanced breast cancer.

In this study, we found no correlation between the timing of adjuvant radiotherapy and risk of relapse. For early stages, delays of more than eight to 12 weeks seem to increase the risk of local relapse in observational studies, but results are conflicting [11]. Moreover, no phase III studies about the optimal interval between surgery and radiotherapy are available. Some data suggest that a delay between surgery and first radiation treatment could be associated with an increased risk of local recurrence and are, therefore, contradictory [12-14]. Huang et al. published a meta-analysis of 10 observational studies with 7401 patients showing that delayed initiation of radiotherapy with a surgery-radiotherapy interval longer than eight weeks was associated with an increase in local recurrence rates at five years (odds ratio, 1.62; 95% CI, 1.21 to 2.16) [15]. Livi et al. showed the timing of postoperative radiotherapy in 4820 patients was not an independent prognostic factor for local recurrence [16]. In patients treated with breast-conserving surgery, local control did not show a difference between three groups [17]. The 15-year local RFS rates were 89.3% for women irradiated <45 days, 83.0% for those irradiated 45 to 56 days (HR, 1.4; 95% CI, 0.7 to 2.6; P=0.360), and 46.4% for patients starting radiotherapy at 57 to 111 days following surgery (HR, 0.7; 95% CI, 0.3 to 1.6; P=0.416) [17]. A German study failed to demonstrate an association between a delay of radiotherapy with decreased local control or overall survival in women treated with or without chemotherapy [18]. An update from the International Breast Cancer Study Group Trials VI and VII detected no significantly increased risk of local failures in patients delaying their radiation therapy until after systemic treatment. A trend of increased risk in local recurrence in patients under 40 years old and nodal status was found [19]. When using adjuvant chemotherapy, several randomized trials tested the optimum sequencing. Dose escalation of chemotherapy schemes prior to radiotherapy frequently resulted in a prolonged surgery-radiotherapy interval. However, these studies were not designed to evaluate the effect of delayed-onset radiotherapy [20-21].

With respect to survival, we found no statistical difference for delays on adjuvant radiotherapy in patients with early breast cancer. However, we did find a statistical decrease in DSS in patients with locally advanced cancer and a delay of 60 days or more before starting radiotherapy (P>0.001). A meta-analysis conducted by Chan et al. indicated a relative risk of 1.11/month of adjuvant radiotherapy delay for local relapse even when chemotherapy was offered, but no significant effect was observed on distant metastasis or overall survival [22]. Overall, the literature shows varying conclusions suggesting that radiotherapy should not be delayed more than 20 weeks after surgery when adjuvant chemotherapy is not administered [23]. Karlsson et al. indicated radiotherapy timing was not significantly associated with the interval to local recurrence, disease-free survival or overall survival in patients receiving adjuvant endocrine therapy for radiotherapy delays of up to 20 weeks [24]. Regarding the impact of delaying radiotherapy until completion of systemic treatment, studies indicate there is no impact on overall survival with chemotherapy administered first; it is suggested that adjuvant radiotherapy should be administered within seven months of surgery [23,25].

Our findings support the importance of avoiding unnecessary delays in initiating radiotherapy. To our knowledge, this the first such study in a Mexican population. Although the sample size of our population was adequate, the major bias of our study was the lack of a randomized design; therefore, it should be interpreted only as a retrospective analysis.

## Conclusions

Results available on relapse and survival are inconclusive, and, in the absence of randomized evidence on the timing of adjuvant treatments, delays in the initiation of radiotherapy should be avoided. Waiting times for radiotherapy should be as short as reasonably achievable, given the specific risk factors in the individual patient.
